# Supplementary material for: Mechanisms governing the pioneering and redistribution capabilities of the non-classical pioneer PU.1
Source: Nat Commun. 2020 Jan 21;11:402. doi: 10.1038/s41467-019-13960-2 (PMC6972792; doi:10.1038/s41467-019-13960-2)
Supplement: Supplementary file 7 — Source data [file 41467_2019_13960_MOESM7_ESM.zip › Source_Data/Figure6/Figure6B_MotifScanOutput/FLI1_PU.1/homerResults/motif22.similar.html]

motif22

## Information for motif22

G
A
T
C
A
C
T
G
G
T
A
C
A
C
T
G
A
C
T
G
C
G
T
A
C
G
T
A
A
C
T
G
C
G
T
A
A
C
T
G
A
C
T
G
A
T
G
C
A
C
T
G
  
Reverse Opposite:  

T
G
A
C
T
A
C
G
A
G
T
C
A
G
T
C
A
C
G
T
A
G
T
C
A
C
G
T
A
C
G
T
A
G
T
C
A
G
T
C
A
C
T
G
T
G
A
C
C
T
A
G
  

|  |  |
| --- | --- |
| p-value: | 1e-55 |
| log p-value: | -1.282e+02 |
| Information Content per bp: | 1.792 |
| Number of Target Sequences with motif | 288.0 |
| Percentage of Target Sequences with motif | 2.89% |
| Number of Background Sequences with motif | 268.9 |
| Percentage of Background Sequences with motif | 0.74% |
| Average Position of motif in Targets | 101.5 +/- 56.1bp |
| Average Position of motif in Background | 107.2 +/- 76.5bp |
| Strand Bias (log2 ratio + to - strand density) | 0.5 |
| Multiplicity (# of sites on avg that occur together) | 1.01 |
| Motif File: | file (matrix) reverse opposite |

### Similar de novo motifs found

|  |  |  |  |  |  |  |  |
| --- | --- | --- | --- | --- | --- | --- | --- |
| Rank | Match Score | Redundant Motif | P-value | log P-value | % of Targets | % of Background | Motif file |
| 1 | 0.763 | T A G C A T G C A T C G C T A G C T G A C G T A C T A G T A C G A T C G A T C G T A G C T A C G T A C G A T C G | 1e-55 | -127.330470 | 5.35% | 2.16% | motif file (matrix) |
